# Dinuclear Lanthanide(III) Complexes from the Use of Methyl 2-Pyridyl Ketoxime: Synthetic, Structural, and Physical Studies

**DOI:** 10.3390/molecules26061622

**Published:** 2021-03-15

**Authors:** Christina D. Polyzou, Helen Nikolaou, Catherine P. Raptopoulou, Konstantis F. Konidaris, Vlasoula Bekiari, Vassilis Psycharis, Spyros P. Perlepes

**Affiliations:** 1 Department of Chemistry, University of Patras, 26504 Patras, Greece; christipoche@gmail.com (C.D.P.); eleni.nikolaou-2@postgrad.manchester.ac.uk (H.N.); 2 Institute of Nanoscience and Nanotechnology, NCSR “Demokritos”, 15310 Aghia Paraskevi Attikis, Greece; c.raptopoulou@inn.demokritos.gr; 3 Department of Pharmacy, University of Patras, 26504 Patras, Greece; konstantis.konidaris@gmail.com; 4 Department of Crop Science, University of Patras, 30200 Messolonghi, Greece; 5 Foundation for Research and Technology-Hellas (FORTH), Institute of Chemical Engineering Sciences (ICE-HT), Platani, B.O. Box 1414, 26504 Patras, Greece

**Keywords:** coordination chemistry, dinuclear lanthanide(III) complexes, magnetic properties of gadolinium(III) complexes, metal complexes of methyl 2-pyridyl ketoxime, photoluminescence studies, single-crystal X-ray structures

## Abstract

The first use of methyl 2-pyridyl ketoxime (mepaoH) in homometallic lanthanide(III) [Ln(III)] chemistry is described. The 1:2 reactions of Ln(NO_3_)_3_·*n*H_2_O (Ln = Nd, Eu, Gd, Tb, Dy; *n* = 5, 6) and mepaoH in MeCN have provided access to complexes [Ln_2_(O_2_CMe)_4_(NO_3_)_2_(mepaoH)_2_] (Ln = Nd, **1**; Ln = Eu, **2**; Ln = Gd, **3**; Ln = Tb, **4**; Ln = Dy, **5**); the acetato ligands derive from the Ln^III^—mediated hydrolysis of MeCN. The 1:1 and 1:2 reactions between Dy(O_2_CMe)_3_·4H_2_O and mepaoH in MeOH/MeCN led to the all-acetato complex [Dy_2_(O_2_CMe)_6_(mepaoH)_2_] (**6**). Treatment of **6** with one equivalent of HNO_3_ gave **5**. The structures of **1**, **5,** and **6** were solved by single-crystal X-ray crystallography. Elemental analyses and IR spectroscopy provide strong evidence that **2**–**4** display similar structural characteristics with **1** and **5**. The structures of **1**–**5** consist of dinuclear molecules in which the two Ln^III^ centers are bridged by two bidentate bridging (*η*^1^:*η*^1^:*μ*_2_) and two chelating-bridging (*η*^1^:*η*^2^:*μ*_2_) acetate groups. The Ln^III^ atoms are each chelated by a *N*,*N*’-bidentate mepaoH ligand and a near-symmetrical bidentate nitrato group. The molecular structure of **6** is similar to that of **5**, the main difference being the presence of two chelating acetato groups in the former instead of the two chelating nitrato groups in the latter. The geometry of the 9-coordinate Ln^III^ centers in **1**, **5** and **6** can be best described as a muffin-type (MFF-9). The 3D lattices of the isomorphous **1** and **5** are built through H-bonding, π⋯π stacking and C-H⋯π interactions, while the 3D architecture of **6** is stabilized by H bonds. The IR spectra of the complexes are discussed in terms of the coordination modes of the organic and inorganic ligands involved. The Eu(III) complex **2** displays a red, metal-ion centered emission in the solid state; the Tb^III^ atom in solid **4** emits light in the same region with the ligand. Magnetic susceptibility studies in the 2.0–300 K range reveal weak antiferromagnetic intramolecular Gd^III…^Gd^III^ exchange interactions in **3**; the *J* value is −0.09(1) cm^−1^ based on the spin Hamiltonian Ĥ = −J(Ŝ_Gd1_·Ŝ_Gd2_).

## 1. Introduction

Oximes are versatile organic molecules containing the >C=N-OH group, which have been extensively used as efficient reagents in analytical chemistry and as excellent ligands in inorganic chemistry. There are several types of oximes: aliphatic and aromatic monoximes derived from ketones r aldehydes, *α*-dioximes, and recently new families of amino/imido oximes and cyanoximes have attracted intense attention [1]. Although the nitroso-oxime tautomerism is known in solution, most solid compounds exist in their oxime form. One of the first uses of oximes in metal chemistry was the gravimetric determination of Ni(II) in the form of the red bis(dimethylglyoximato)nickel(II) [2], an experiment performed by undergraduate Chemistry students in most universities. Today, oxime and oximato metal complexes are central “players” in several aspects of coordination [3,4,5] and bioinorganic [6,7] chemistry, molecular magnetism [8,9,10,11], catalysis [12,13], and in the area of the reactivity of coordinated ligands [14,15,16].

**Figure 1:**
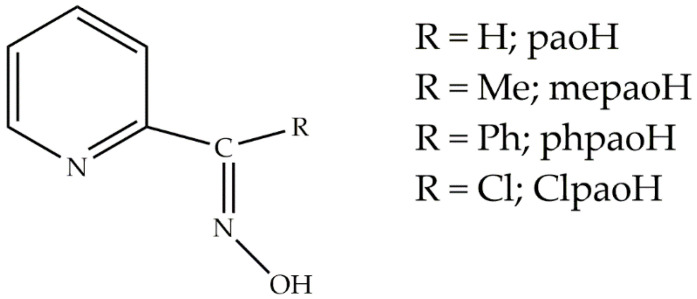
General structural formula of *simple* 2-pyridyl oximes, in which R is a non-donor group; the ligand used in the present work is methyl 2-pyridyl ketoxime (R = Me; mepaoH).

A central theme in our research groups is the study of the coordination chemistry of 2-pyridyl oximes in which the substituent R at the oxime carbon is either a non-donor group (Figure 1) or a donor group (e.g., R = CN, NH_2_, 2-pyridyl, etc.) [17]. Restricting further discussion to the former family of ligands, the emphasis of our efforts (only selected references from our work are given below) has been on (a) the optical and magnetic properties of the resulting homo- and heterometallic dinuclear [18,19,20,21] and polynuclear [22,23,24,25,26,27] complexes, (b) the metal-mediated/promoted/assisted reactivity of these ligands [28,29,30], and (c) the modeling of the solvent extraction of toxic metal ions from aqueous solutions by 2-pyridyl ketoximes [31,32]. A literature survey reveals that a plethora of homometallic complexes of paoH, mepaoH, phpaoH, and ClpaoH (Figure 1), in their neutral or anionic forms, have been prepared and studied with transition [22,23,24,25,27,28,29,31,32] and actinide [21] metal ions. In contrast, with the exception of [LnCl_3_(phpaoH)_3_] (Ln = Nd [33], Sm [34]), such complexes are not known in lanthanide (Ln) chemistry.

We have embarked on a new program aiming to fill the above-mentioned literature gap and prepare 4f-metal complexes with the ligands (neutral or deprotonated) shown in Figure 1. In this work, which is the first of a series of papers on this topic, we report our preliminary results on the reactions of Ln(III) sources and methyl 2-pyridyl ketoxime (mepaoH; R = Me in Figure 1) in the absence of an external base. Our efforts have led to dinuclear Ln(III) complexes with interesting structural features and properties; a point of interest is that the bridging acetato ligands in most of the complexes have resulted from the Ln^III^-assisted hydrolysis of the MeCN (solvent). This paper can also be considered as a continuation of the interest of our groups in the chemistry and properties (photophysical, magnetic, catalytic) of the Ln^III^_2_ complexes [35,36,37,38,39,40,41,42,43,44]; such complexes can potentially combine both optical and magnetic properties within the same molecule leading to luminescent SMMs [45,46]. Ln^III^_2_ compounds continue to attract the intense attention of many inorganic chemistry groups around the world because they are ideal model systems for providing answers to fundamental questions regarding the origin of a certain physical, spectroscopic, or catalytic property, i.e., if such properties arise from the whole molecule (two Ln^III^ centers) or half of it (a single Ln^III^ ion) [47].

## 2. Results and Discussion

### 2.1. Synthetic Comments

A variety of Ln(III)/X^−^/mepaoH reaction systems involving various metal ions and X^−^ (X^−^ = Cl^−^, NO_3_^−^, MeCO_2_^−^), and different solvent media, reagent ratios, and crystallization methods were systematically employed before arriving at the optimized procedures reported in Section 3. Since we were interested in obtaining products with the neutral oxime ligand, we avoided the addition of an external base, e.g., Et_3_N, LiOH·H_2_O, and Me_4_NOH·5H_2_O. Crystalline products were obtained only with X^−^ = NO_3_^−^, MeCO_2_^−^ and representative complexes are described in this work. In the case of X^−^ = Cl^−^, powders with the empirical formulae (evidence from microanalyses) {LnCl_3_(mepaoH)_3_·*n*EtOH} (*n* = 1–2) were obtained from the reactions of hydrated lanthanoid chlorides and mepaoH in 1:3 reaction ratios in refluxing EtOH. We could not obtain single crystals for X-ray structural determination and so we did not pursue this chemistry further.

The 1:2 reactions between Ln(NO_3_)_3_·*n*H_2_O (*n* = 5 or 6) and mepaoH in MeCN gave almost colorless pale-colored (with the color of Ln^III^) or colorless solutions from which crystals (in the case of Nd^III^ and Dy^III^) or crystalline solids (in the case of Eu^III^, Gd^III^, and Tb^III^) of [Ln_2_(O_2_CMe)_4_(NO_3_)_2_(mepaoH)_2_] (Ln = Nd, **1**; Ln = Eu, **2**; Ln = Gd, **3**; Ln = Tb, **4**; Ln = Dy, **5**) were subsequently isolated in moderate yields (30–40% based on the Ln^III^ used). The stoichiometric 1:1 reactions gave again **1**–**5**, albeit in lower yields (~25%). Given the absence of acetate ions from the reaction mixtures, the presence of acetato ligands in the products was surprising to us. The only source of coordinated MeCO_2_^−^ groups in **1**–**5** could be the solvent used (MeCN), which contains 1–2% of H_2_O. Assuming that the complexes are the only products from their respective reaction systems, and that the H_2_O responsible for the generation of acetates comes from the hydrated lanthanide(III) nitrates, the formation of the products can be summarized by the simplified Equation (1); *n* = 6 for Nd(III), Eu(III), Gd(III), Tb(III), and 5 for Dy(III). As mentioned above, the H_2_O present in the solvent can also contribute to the formation of the MeCO_2_^−^ ligands:

(1) $$\begin{array}{ll} 2\;\mathrm{Ln}{\left({\mathrm{NO}}_{3}\right)}_{3}\cdot nH_{2}O & +2\;\mathrm{mepaoH}\;+\;4\;\mathrm{MeCN}\;\overset{\mathrm{MeCN}}{\to }\;\left[{\mathrm{Ln}}_{2}{\left(O_{2}\mathrm{CMe}\right)}_{4}{\left({\mathrm{NO}}_{3}\right)}_{2}{\left(\mathrm{mepaoH}\right)}_{2}\right]+\;4{\;\mathrm{NH}}_{4}\left({\mathrm{NO}}_{3}\right)\\ & +\left(2n-8\right)\;H_{2}O \end{array}$$

In accordance with our belief that Equation (1) represents the real chemistry of the reactions: (i) using more concentrated reaction solutions to increase the yields, the products were contaminated (IR and microanalyses evidences) by variable amounts of precipitated NH_4_(NO_3_); the H_2_O present in the mixtures of the optimized reactions (Section 3) is enough to keep the NH_4_(NO_3_) byproduct soluble, and (ii) employment of distilled MeCN as solvent leads to lower yields and serious contamination of the products by ammonium nitrate.

**Figure 2:**
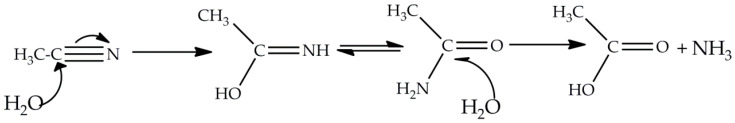
The hydrolysis of MeCN to generate MeCO_2_H.

It is well known in organic chemistry that nitriles can be hydrolyzed to give either amides or carboxylic acids [48,49]. The initially produced hydroxylimine may tautomerize to an amide, which in turn generates a carboxylic acid upon further hydrolysis [48,50]. For MeCN, the two hydrolysis steps are illustrated in Figure 2. Several metal ion-mediated processes for the hydration of nitriles with selective formation of metal-bound carboxamides are known [27,51,52,53], but most of the systems are not catalytic and only a few of them are able to hydrate R’-C≡N under homogeneous catalytic conditions [13,53], usually exhibiting a low activity [53]. The second step of the metal-ion mediated hydrolysis, i.e., conversion to carboxylic acids and ammonia, has been observed only in few cases involving Pt(IV), Os(IV), and Nb(V) systems [53]. To the best of our knowledge, the present work represents the second case in which an Ln(III)-mediated hydrolysis of MeCN is experimentally observed. The first case involved the reactions of hydrated lanthanide(III) nitrates with 2-(4-carboxyphenyl)imidazo[4,5-*f*][1–10]phenanthroline (Hcpip) and 2-phenylimidazo [4,5-*f*][1–10]phenanthroline (pip) in MeCN/H_2_O under solvothermal conditions (~190 °C), which gave the 1D coordination polymers {[Ln_2_(O_2_CMe)_4_(cpip)_2_]}_n_ (Ln = Sm, Eu) and the dinuclear complexes [Ln_2_(O_2_CMe)_6_(pip)_2_], respectively [54]. In the present case, the produced MeCO_2_H is neutralized by the also generated ammonia giving the acetato ligands and providing ammonium cations that provide charge balance for the released nitrate ions, Equation (1). 

Having established the composition of **1**–**5**, we wondered if we could obtain dinuclear Ln(III)/mepaoH complexes with six acetato ligands, i.e., without nitrato groups. Two procedures were investigated: (i) the reaction mixtures that lead to **1**–**5** were refluxed overnight, and (b) complexes **1**–**5** were treated with an excess (3–5 equivs per equiv of dinuclear complex) of an equimolar mixture of MeCO_2_H and Et_3_N in MeCN; in both cases, complexes [Ln_2_(O_2_CMe)_4_(NO_3_)_2_(mepaoH)_2_] were again isolated, i.e., no reaction occurred. In a modification of the second route, the 1:6:6:2 Ln(NO_3_)_3_·*n*H_2_O/MeCO_2_H/Et_3_N/mepaoH reaction mixtures in MeCN gave again complexes **1**–**5** in moderate yields (25–35% based on the Ln^III^ available), Equation (2). This is strong evidence that the mixed acetato/nitrato complexes are the thermodynamically preferred products from the Ln^III^/NO_3_^−^/MeCO_2_^−^/mepaoH reaction systems in MeCN:

(2) $$\begin{array}{ll} 2\;\mathrm{Ln}{\left({\mathrm{NO}}_{3}\right)}_{3}\cdot nH_{2}O & +2\;\mathrm{mepaoH}\;+\;4{\;\mathrm{MeCO}}_{2}H+4{\;\mathrm{Et}}_{3}N\overset{\mathrm{MeCN}}{\to }\;\left[{\mathrm{Ln}}_{2}{\left(O_{2}\mathrm{CMe}\right)}_{4}{\left({\mathrm{NO}}_{3}\right)}_{2}{\left(\mathrm{mepaoH}\right)}_{2}\right]\\ & +\;4\;\left({\mathrm{Et}}_{3}\mathrm{NH}\right)\left({\mathrm{NO}}_{3}\right)+2n{\;H}_{2}O \end{array}$$

The obvious route to prepare all-acetato dinuclear Ln(III)/mepaoH complexes was to avoid the presence of NO_3_^−^ ions in the reaction systems. This was examined in the case of Dy(III). Thus, the refluxing 1:1 or 1:2 reactions between Dy(O_2_CMe)_3_·4H_2_O and mepaoH in a solvent mixture of MeOH/MeCN (1:2 *v*/*v*) led to a slurry, the filtration of which gave an almost colorless solution from which colorless crystals of [Dy_2_(O_2_CMe)_6_(mepaoH)_2_] (**6**) were subsequently isolated in good yield (50–60% based on Dy^III^), Equation (3). MeOH was necessary to keep an amount of Dy(O_2_CMe)_3_·4H_2_O (insoluble in MeCN) soluble in order to react with the ligand; the reaction in MeOH and subsequent precipitation with Et_2_O gave a powder, most probably (IR evidence) a mixture of **6** and Dy(O_2_CMe)_3_·4H_2_O. Treatment of **6** with 2–2.5 equivs of HNO_3_ (from a 2N solution) in MeCN gave complex **5**, Equation (4), emphasizing again the thermodynamic stability of the mixed acetato/nitrato complexes. Excess of 2N HNO_3_ leads to the decomposition of the product:

(3) $$2\;\mathrm{Dy}{\left(O_{2}\mathrm{CMe}\right)}_{3}\cdot 4H_{2}O+2\;\mathrm{mepaoH}\;\overset{\mathrm{MeOH}/\mathrm{MeCN}}{\to }\left[{\mathrm{Dy}}_{2}{\left(O_{2}\mathrm{CMe}\right)}_{6}{\left(\mathrm{mepaoH}\right)}_{2}\right]+\;8\;H_{2}O$$

(4) $$\left[{\mathrm{Dy}}_{2}{\left(O_{2}\mathrm{CMe}\right)}_{6}{\left(\mathrm{mepaoH}\right)}_{2}\right]+\;8\;{\mathrm{HNO}}_{3}\overset{\mathrm{MeCN}}{\to }\;\left[{\mathrm{Dy}}_{2}{\left(O_{2}\mathrm{CMe}\right)}_{4}{\left({\mathrm{NO}}_{3}\right)}_{2}{\left(\mathrm{mepaoH}\right)}_{2}\right]+2{\;\mathrm{MeCO}}_{2}H$$

### 2.2. Description of Structures

**Figure 3:**
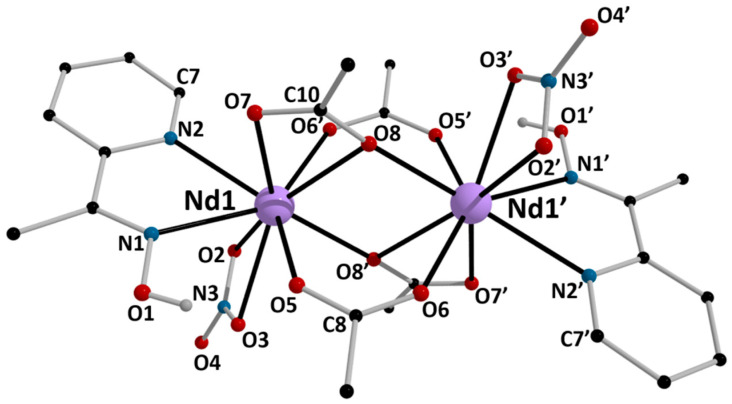
Partially labeled plot of the structure of the dinuclear molecule [Nd_2_(O_2_CMe)_4_(NO_3_)_2_(mepaoH)_2_] that is present in the crystal of **1**. Symmetry operation: (‘) = –*x*+2, −*y*+1, –*z*+2.

**Figure 4:**
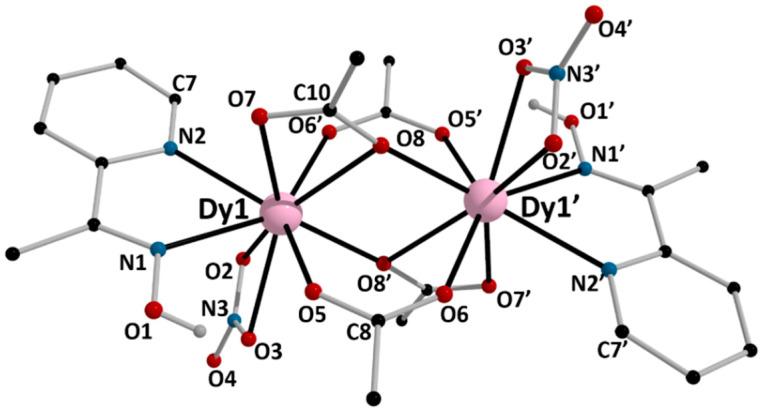
Partially labeled plot of the structure of the dinuclear molecule [Dy_2_(O_2_CMe)_4_(NO_3_)_2_(mepaoH)_2_] that is present in the crystal of **5**. Symmetry operation: (‘) = –*x*+2, −*y*+1, –*z*+2.

**Figure 5:**
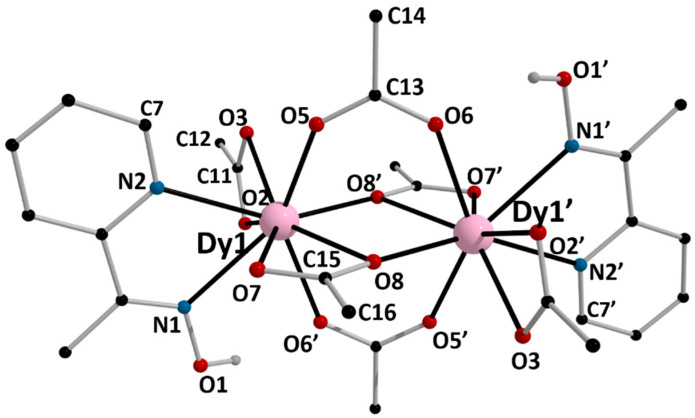
Partially labeled plot of the structure of the dinuclear molecule [Dy_2_(O_2_CMe)_6_(mepaoH)_2_] that is present in the crystal of **6**. Symmetry operation: (‘) = −*x*, −*y*, −*z*.

**Figure 6:**
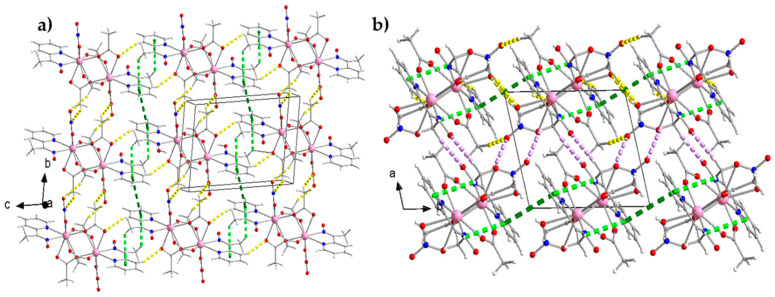
(**a**) 2D arrangement of the isomorphous complexes **1** and **5** parallel to the (100) plane. Thick dashed yellow lines indicate the C4-H(C4)⋯O7 (2.49 Å), C9-H_B_(C_g_)⋯O4 (2.43 Å) and C11-H_B_(C11)⋯O2 (2.60 Å) H bonds, where C4 is an aromatic carbon atom of the mepaoH ligand and C9, C11 are the methyl carbon atoms of the crystallographically independent acetato groups. Thick dashed light green and dark green lines represent the C-H⋯π and π⋯π interactions, respectively, described in the text; (**b**) stacking of layers parallel to the *a* crystallographic axis for **1** and **5**. Thick dashed violet lines represent the C1-H_A_(C1)⋯O1 (2.52 Å) and C1-H_B_(C1)⋯O4 (2.59 Å) H bonds, where C1 is the methyl carbon atom of the mepaoH ligand. All the distance and angle parameters for the H bonds, as well as the symmetry operations are listed in Table S3.

**Figure 7:**
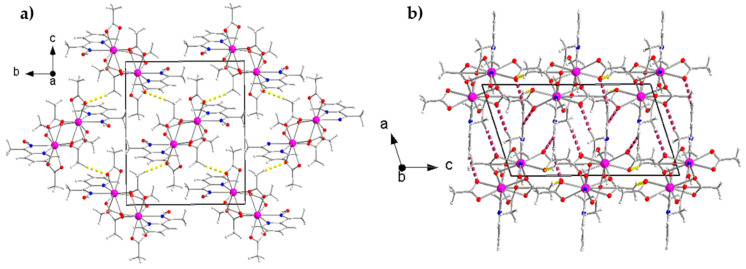
Intermolecular interactions in the crystal structure of complex **6**. (**a**) 2D arrangement of the dinuclear molecules parallel to the (100) plane. Thick dashed yellow lines indicate the C16-Hc(C16)⋯O2 (2.46 Å) H bond, where C16 is the methyl carbon atom of the crystallographically unique *η*^1^*:η*^2^*:μ*_2_ (2.12) acetato group; (**b**) stacking of layers parallel to the *α* crystallographic axis. Thick dashed red-violet lines represent the C4-H(C4)⋯O3 (2.35 Å), C6-H(C6)⋯O2 (2.44 Å) and C14-H_C_(C14)⋯O7 (2.58 Å) H bonds; C4 and C6 are aromatic carbon atoms of the mepaoH ligand. The distances and angles for the H bonds, as well as the symmetry operations are listed in Table S4.

The structures of **1**, **5,** and **6** were determined by single-crystal X-ray crystallography. Crystallographic data are listed in the Supplementary Table S1. Various structural plots are shown in Figure 3, Figure 4, Figure 5, Figure 6 and Figure 7 and Supplementary Figures S1–S3. Selected interatomic distances and bond angles are given in Table 1 and Table 2. CShM values for the potential coordination polyhedra of the Ln^III^ centers in **1**, **5,** and **6** are summarized in Table S2, while H-bonding interactions for the three structures are listed in Tables S3 and S4.

Complexes **1** and **5** crystallize in the triclinic space group *P*ī and are isomorphous; thus, a general description will be given (Ln = Nd, Dy). The structures consist of dinuclear molecules [Ln_2_(O_2_CMe)_4_(NO_3_)_2_(mepaoH)_2_]. There is a crystallographically imposed inversion center at the midpoint of the Ln⋯Ln1′ vector. The Ln^III^ atoms are each chelated by a *N*,*N*’-bidentate mepaoH ligand (*η*^2^ or 1.011 using Harris notation [55]) and an almost symmetrical bidentate nitrato group (*η*^2^ or 1.110). The metal centers are bridged by two pairs of acetato ligands. One of them consists of symmetrical *syn, syn η*^1^*:η*^1^*:μ*_2_ (or 2.11) groups, the Ln1-O5 and Ln1-O6′ bond lengths being 2.426(5) and 2.404(4) Å, respectively, for **1**, and 2.343(7) and 2.312(8) Å, for **5**, respectively. The second pair of bridging acetato groups are best described as chelating-bridging (*η*^1^*:η*^2^*:μ*_2_ or 2.12), since, in addition to the two typical Ln1-O bonds [Nd1-O7 = 2.503(5) Å, Nd1-O8′ = 2.385(5) Å and Dy1-O7 = 2.421(8) Å, Dy1-O8′ = 2.281(7) Å], there is now a weaker Ln1-O bond [Nd1-O8 = 2.578(4) Å and Dy1-O8 = 2.522(8) Å]. Considering only the monoatomic O-bridges (O8, O8′), the strictly planar (by symmetry) core of the complexes is {Ln^III^_2_(*μ*_2_-OR’)_2_}^4+^, where RO’^−^ = MeCO_2_^−^. The Nd1⋯Nd1′ [3.877(1) Å] and Dy1⋯Dy1′ [3.795(1) Å] distances are short due to the quadruplicate bridge. The Ln1-O8/O8′-Ln1′ angles are 102.7(2) and 104.3(3)^o^ for **1** and **5**, respectively. The nitrato N-O bond lengths involving the “free” oxygen atom are slightly shorter to those involving the coordinated oxygen atoms; for example, the N3-O4 bond length in **5** is 1.230(13) Å, while the N3-O2 and N3-O3 bond distances are 1.289(15) and 1.266(13) Å, respectively. The presence of monoatomic oxygen bridges in the 2.12 acetato ligands results in a significant difference between the lengths of the two carboxylate C-O bonds, the C10-O8 distance [1.289(9) Å for **1** and 1.293(12) Å for **5** being longer than the C10-O7 distance [1.227(8) Å for **1** and 1.236(13) Å for **5**]. For a given bond, the Ln-O/N bond lengths follow the Nd>Dy order (Table 1) due to lanthanide contraction; this trend is also observed for the Ln^III…^Ln^III^ distances. The Ln-O/N bond lengths in **1** and **5** are typical for 9-coordinate Nd(III) [33,56,57] and Dy(III) [35,38,41,54,58,59,60,61,62]. The Ln1/Ln1′ coordination geometry was evaluated using the program SHAPE [61,63] and is best described as muffin-type (Figure S1, Table S2); an alternative description of the polyhedron could be that of a spherical-capped square antiprism.

There are two crystallographically independent H bonds within the dinuclear molecules (intramolecular H bonds, Figure S2 and Table S3). The donors are the oxime oxygen atoms (O1/O1′) and the aromatic carbon atoms C7/C7′, and the acceptors are the coordinated acetato oxygen atoms O5/O5′ and O6′/O6, respectively. Given the rather small donor-H⋯acceptors angles, these bonds can be considered as weak.

Complex **6** crystallizes in the monoclinic space group *P*2_1_/*c*. Although its composition is different compared with that of **5** (and **1**), the molecular structures are similar. The only significant difference is the presence of the two chelating acetato groups (*η*^2^ or 1.11) in **6** instead of the two chelating nitrato groups (*η*^2^ or 1.110) in **5**. Again, the two Dy^III^ centers are linked by four MeCO_2_^−^ ligands. Two of them bridge in the classical *syn, syn η*^1^*:η*^1^*:μ*_2_ (or 2.11) fashion, and the other two bridge in the less common *η*^1^*:η*^2^*:μ*_2_ (2.12) mode; the latter have an oxygen atom (O7, O7′) bound terminally to one metal ion, and the oxygen atom (O8, O8′) bound in a *μ*_2_ manner to both Dy^III^ atoms, forming a monoatomic bridge. One chelating 1.011 mepaoH molecule and one chelating (1.11) MeCO_2_^−^ ion complete 9-coordination at each metal center. Due to the inversion center, the Dy1O8Dy1′O8′ core is perfectly planar. The coordination polyhedron of Dy1/Dy1′ is best described as muffin (Figure S1, Table S2). The interatomic distances and bond angles in **6** and **5** are similar; a notable difference is that the Dy-N_oxime_ bond length [2.593(8) Å] is larger than that of the Dy-N_pyridyl_ bond length [2.525(7) Å] in **6**, while the opposite trend [2.510(11) vs. 2.535(9) Å, respectively] is observed in **5**.

There are two crystallographically independent, weak intramolecular H bonds in **6** (Figure S3 and Table S4). The donors are the oxime oxygen atoms (O1/O1′) and the aromatic carbon atoms C7/C7′, and the acceptors are the coordinated acetato oxygen atoms O6′/O6 and O5/O5′, respectively.

At the supramolecular level, the crystal structures of the isomorphous complexes **1** and **5** are stabilized by intermolecular H bonds (Table S3), π–π overlaps and C-H⋯π interactions. The distance between the centrosymmetrically-related pyridyl rings N2C3C4C5C6C7 and N2*C3*C4*C5*C6*C7* [(*) = −*x*+2, −*y*, −*z*+1] is 3.74(3) Å for **1** and 3.73(5) Å for **5**. The C-H⋯π interactions are evaluated using the Hc(C1)⋯C_g_1** distance, which is 3.068(1) Å for **1** and 2.910(1) Å for **5** [(**) = = −*x*+2, −*y*+1, −*z*+1]; C_g_1 is the centroid of the pyridyl ring of mepaoH, and Hc(C1) is one of the methyl hydrogen atoms of mepaoH. Thus, layers of molecules are formed parallel to the (100) plane (Figure 6a). The layers interact through H bonds and are stacked along the *a* crystallographic axis, thus building the 3D architectures (Figure 6b).

The crystal structure of **6** is built through H bonds (Table S4). The dinuclear molecules form layers parallel to the (100) plane (Figure 7a), which interact further to create the 3D architecture of the structure (Figure 7b).

Complexes **1**, **5,** and **6** are members of a rather large family of dinuclear complexes containing the {Ln_2_(*η*^1^:*η*^1^:*μ*_2_-O_2_CMe)_2_(*η*^1^:*η*^2^:*μ*_2_-O_2_CMe)_2_}^2+^ unit [39,54,56,57,58,59,60,64,65,66]; (only representative references are given), most of which [54,58,60,64,65] contain an extra chelating acetato group per Ln^III^ center; complexes containing a chelating nitrato group per Ln^III^ atom, i.e., the unit {Ln_2_(*η*^1^:*η*^1^:*μ*_2_-O_2_CMe)_2_(*η*^1^:*η*^2^:*μ*_2_-O_2_CMe)_2_-(*η*^2^-OOΝO)}, are few [39,56,59,66].

### 2.3. Characterization of Selected Complexes

In the solid-state (KBr) IR spectra of **1**–**6**, the presence of a medium-intensity band at ~3250 cm^−1^ is assigned to the *v*(OH) vibration of the mepaoH ligand [18,21]; the broadness and the relatively low wavenumber of the band are both indicative of H-bonding interactions established by crystallography (*vide supra*). The medium-intensity band at 1116 cm^−1^ in the spectrum of free mepaoH has been assigned to the *v*(OH)_oxime_ mode [21]; this band has been shifted to lower wavenumber (~1090 cm^−1^) in the spectra of the six complexes due to coordination of the oxime nitrogen [21]. The in-plane pyridyl deformation of free mepaoH at 632 cm^−1^ shifts upwards in the spectra of the complexes (~685 cm^−1^) confirming the involvement of the ring N-atom in coordination [18,24,32]. The spectra of **1**–**5** are almost identical supporting similar structures of the complexes [Ln_2_(O_2_CMe)_4_(NO_3_)_2_(mepaoH)_2_] (Ln = Nd, Eu, Gd, Tb, Dy). Many bands appear in the 1370–1630 cm^−1^ region in the IR spectra of **1**–**6**. Contributions from the *δ*(OH)_oxime_, *v*(C=N)_oxime_, *v*_as_(CO_2_), *v*_s_(CO_2_), aromatic ring stretching vibrations and nitrato stretching modes (for **1–5**) would be expected in this region, rendering exact assignments and discussion of the coordination shifts rather impossible. The bands at 1500, 1295, and ~960 cm^−1^ in **1**–**5** are assigned to the *v*_1_(A_1_)[*v*(N=O], *v*_5_(B_2_)[*v*_as_(NO_2_)] and *v*_2_(A_1_)[*v*_s_(NO_2_)] vibrational modes, respectively, of the nitrato group [67]; the separation of the two highest-wavenumber stretching bands is large (~200 cm^−1^) and typical for bidentate chelating (*C*_2v_) nitrato groups [67]. These three bands do not appear in the spectrum of the nitrate-free compound **6**.

**Figure 8:**
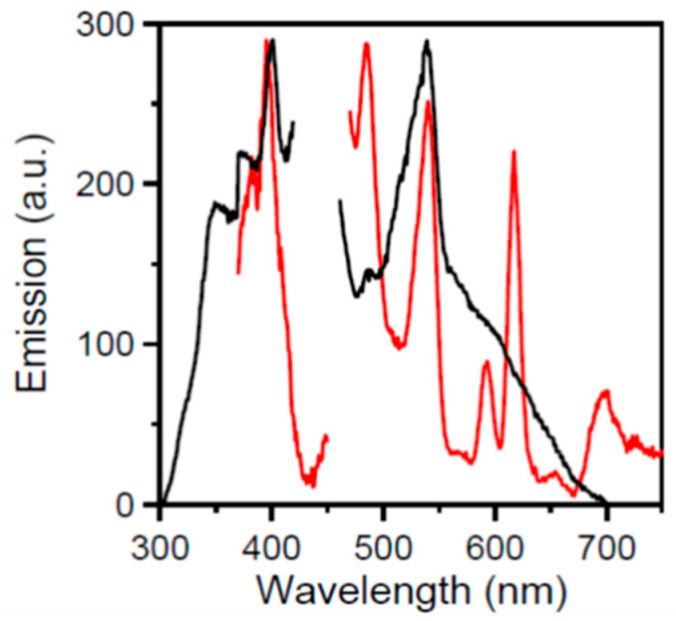
Solid-state, room-temperature excitation (left) and emission (right) spectra of mepaoH (black curves) and complex **2** (red curves). The emission spectra were detected at 397 nm for both compounds. The excitation spectra were detected with maximum emission at 545 nm for mepaoH and 618 nm for **2**.

**Figure 9:**
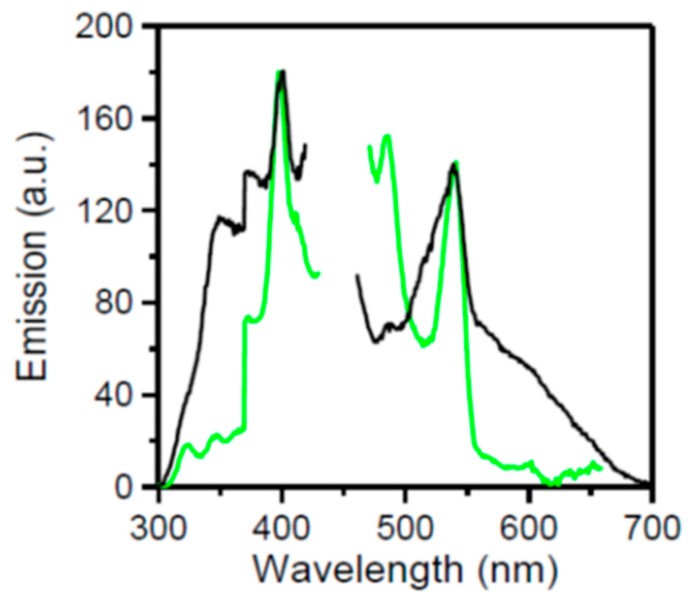
Solid-state, room-temperature excitation (left, emission, at 545) and emission (right, excitation at 397 nm) of mepaoH (black curves) and complex **4** (green curves).

The luminescence of Ln^III^ ions results from electronic transitions within the partially filled 4f^n^ orbitals. Due to the intrinsically low probability of such electronic 4f-4f transitions, the photon absorption cross section and the emission brightness are small. A common approach to overcome this disadvantage is to take advantage of a sensitizing entity (usually an organic ligand) that can effectively harvest the excitation energy and transfer it to the Ln^III^ ion. An ideal organic ligand for this purpose should: (i) feature a large photon absorption cross section; and (ii) exhibit an energy level scheme that allows for an effective transfer of the absorbed energy to the Ln^III^ center [68,69,70,71]. Since Eu(III) and Tb(III) complexes often emit light in the visible region of the electromagnetic spectrum, we investigated the photoluminescence properties of complexes **2** and **4** (Figure 8 and Figure 9, respectively).

The free mepaoH ligand emits with a maximum at ~540 nm, upon maximum excitation at 397 nm. Upon the same maximum excitation (397 nm), the solid complex [Eu_2_(O_2_CMe)_4_(NO_3_)_2_(mepaoH)_2_] (**2**) displays emission peaks at 593, 618, 653, and 695 nm due to Eu^III^ [41,68,69]. These peaks are assigned to the characteristic ^5^*D*_0_→^7^*F*_j_ (j = 0–4) transitions of this metal ion. Specific assignments [41,43] are as follows: ^5^*D*_0_→^7^*F*_0,1_ (593 nm); ^5^*D*_0_→^7^*F*_2_ (618 nm); ^5^*D*_0_→^7^*F*_3_ (653 nm); ^5^*D*_0_→^7^*F*_4_ (695 nm). The dominant peak is due to the hypersensitive ^5^*D*_0_→^7^*F*_2_ transition. The higher intensity of this transition compared with that of the magnetic-dipole allowing ^5^*D*_0_→^7^*F*_1_ transition indicates that this complex has a structure with no imposed symmetry at Eu^III^ (the molecules of **2**, which are proposed to be isostructural with those of the crystallographically characterized **1** and **5**, have an inversion center at the midpoint of the Ln^III…^Ln^III^ vector). Generally, complexes with a centrosymmetric coordination sphere of Eu^III^ (which is not the case in **2**) have a (^5^*D*_0_→^7^*F*_2_)/(^5^*D*_0_→^7^*F*_1_) intensity ratio lower than 0.7 [41]. The lifetime of the excited state of Eu(III) was found to be 0.68 ms, a value typical for Ln(III) complexes [43,71].

The emission peaks at ~545 and ~490 nm arise from the coordinated mepaoH ligand, since the free ligand (black curve in Figure 8) emits in this area with maxima at approximately these wavelengths (the slight shifts are due to coordination) under identical excitation conditions.

In the case of solid **4**, the detected emission spectrum seems to arise mainly from the mepaoH ligand and not from Tb^III^; this was rather expected since Tb^III^ ions’ characteristic emission peaks are in the same spectral area [41,72].

Complexes of some Ln^III^ ions are currently protagonists in several areas of molecular magnetism [71,73,74,75,76,77,78,79,80,81,82,83]. The paramagnetic nature of most Ln^III^ ions arises from their large number of unpaired 4f electrons (large ground-state spin). This property combined with the magnetic anisotropy of some complexes can lead to Single-Molecule Magnetism (SMM) behavior [76,77,78,79,80,81,82,83]. Single-Molecule Magnets (SMMs) are today at the forefront of new technological advances, e.g., in quantum information processing and spintronics [71]. The goal of this work is not associated with the study of the magnetic properties of Ln(III)/mepaoH complexes. A full magnetic study of ~20 carboxylato-bridged dinuclear Tb(III), Dy(III), Ho(III), Er(III), and Yb(III) complexes with the four capping ligands shown in Figure 1, including the field-induced SMM behavior of some of them, will be reported in a separate work (currently in preparation). Here, we simply describe the magnetic properties of **3**, which contains Gd^III^ centers that present no spin-orbit coupling at the first order.

**Figure 10:**
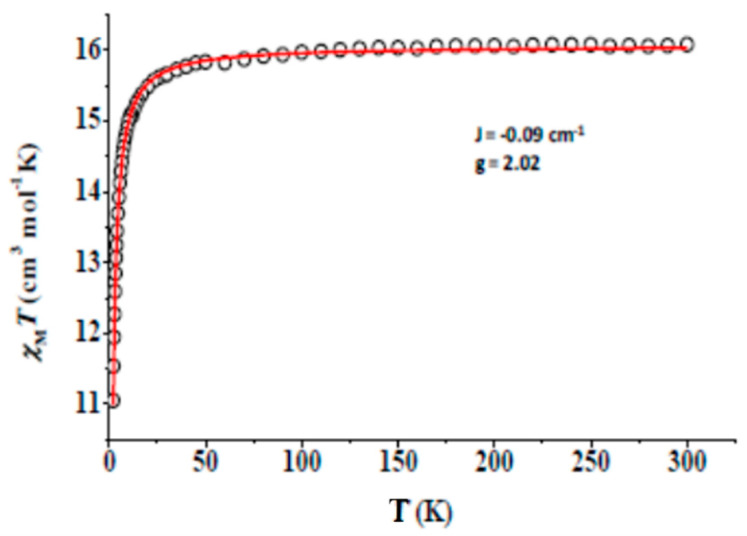
Temperature dependence of the *χ*_Μ_*Τ* product of complex **3** at 0.1 T. The solid red line is the fit of the data to the theoretical Heisenberg model for a Gd^III^_2_ complex; see the text for details.

Direct-current (dc) molar magnetic susceptibility (*χ*_Μ_) data on a polycrystalline sample of **3** were collected in the 2.0–300 K range using an applied field of 0.1 T; the data are presented in the *χ*_Μ_*Τ* vs. *T* plot in Figure 10, where *T* is the absolute temperature. At room temperature, the *χ*_Μ_*Τ* value of **3** is 16.02 cm^3^·K·mol^−1^, in very good agreement with the expected theoretical value of 15.75 cm^3^·K·mol^−1^ for two noninteracting Gd^III^ centers (*S* = 7/2, *L* = 0, *g* = 2.00). The value of *χ*_Μ_*Τ* remains almost constant in the 50–300 K range and then decreases rapidly with decreasing *T* to reach a value of 11.05 cm^3^·K·mol^−1^ at 2.0 K; this decrease directly reveals the presence of weak antiferromagnetic, intramolecular Gd^III…^Gd^III^ exchange interactions. The experimental data were fitted to the van Vleck analytical expression of the susceptibility derived from the isotropic Heisenberg spin Hamiltonian of Equation (5). The best-fit parameters obtained are *J* = −0.09 (1) cm^−1^ and *g* = 2.02. Because of the little overlap between the shielded 4f orbitals and the orbitals of the bridging oxygen atoms, the observed antiferromagnetic exchange interaction is very weak. Such small values have been observed in systems containing symmetrically bridged {Gd^III^_2_(*μ*_2_-OR’’)_2_} cores (R’’ = various groups) [38,40,42,84,85,86].

(5) $$\hat{H}=-J\left({\hat{S}}_{Gd1}\cdot {\hat{S}}_{Gd2}\right)$$

## 3. Experimental Section

### 3.1. Materials, Physical and Spectroscopic Measurements

All manipulations were performed under aerobic conditions. Reagents and solvents were purchased from Alfa Aesar (Karlsruhe, Germany) and Sigma-Aldrich (Tanfrichen, Germany), and used as received. The free ligand methyl 2-pyridyl ketoxime (mepaoH; R = Me in Figure 1) was synthesized as described in the literature [87] in >70% yields; its purity was checked by ^1^H NMR spectroscopy and the determination of its melting point (found, 118–119 °C; reported, 121 °C).

Elemental analyses (C, H, N) were performed by the University of Patras Center of Instrumental Analysis. FT-IR spectra (4000–450 cm^−1^) were recorded using a PerkinElmer 16PC spectrometer (PerkinElmer, Watham, MA, USA) with samples prepared as KBr pellets under pressure. Solid-state emission and excitation spectra were recorded at room temperature using a Cary Eclipse fluorescence spectrometer (Varian, Palo Alto, CA, USA). Variable-temperature, solid-state direct current (dc) magnetic susceptibility data in the 2.0–300 K range were collected on a Quantum Design MPMS-XL SQUID magnetometer (Quantum Design, San Diego, CA, USA) operating at 0.1 T housed at the University of Crete. The magnetic data were corrected for the intrinsic diamagnetic contributions using Pascal’s constants [88].

### 3.2. Synthesis of the Representative Complex [Nd_2_(O_2_CMe)_4_(NO_3_)_2_(mepaoH)_2_] (***1***)

Method (a): To a stirred colorless solution of mepaoH (0.136 g, 1.00 mmol) in MeCN (10 mL), solid Nd(NO_3_)_3_·6H_2_O (0.219 g, 0.50 mmol) was added. The solid soon dissolved and the resulting pale lilac solution was stirred for a further 30 min, and filtered and stored in a closed flask at room temperature. X-ray quality, almost colorless crystals of the product, were precipitated within a period of one week. The crystals were collected by filtration, washed with cold MeCN (2 × 1 mL) and Et_2_O (3 × 2 mL), and dried in air overnight, Yield: 34% (based on the Nd^III^ available). Anal. Calcd. (%) for C_22_H_28_Nd_2_N_6_O_16_: C, 28.69; H, 3.07; N, 9.13. Found (%): C, 28.81; H, 2.99; N, 8.93. Selected IR data (KBr, cm^−1^): 3260 mb, 1590 s, 1492 s, 1420 s, 1295 s, 1130 m, 1090 m, 1020 s, 960 m, 791 s, 682 s, 410 m.

Method (b): To a stirred colorless solution containing mepaoH (0.136 g, 1.00 mmol), glacial MeCO_2_H (0.173 mL, 3.00 mmol) and Et_3_N (0.390 mL, 3.00 mmol) in MeCN (15 mL), solid Nd(NO_3_)_3_·6H_2_O (0.219 g, 0.50 mmol) was added. The solid soon dissolved and the resulting pale lilac solution was stirred for a further 30 min, and filtered and stored in a closed flask at room temperature. X-ray, almost colorless crystals were precipitated within a period of 4–5 d. The crystals were collected by filtration, washed with cold MeCN (4 × 2 mL) and Et_2_O (4 × 2 mL), and dried in air overnight. Yield: 27% (based on the Nd^III^ available). Anal. Calcd. (%) for C_22_H_28_Nd_2_N_6_O_16_: C, 28.69; H, 3.07; N, 9.13. Found (%): C, 28.43; H, 3.17; N, 8.99. The IR spectrum of the solid sample was identical with the authentic sample obtained by method (a).

### 3.3. Syntheses of the Complexes [Eu_2_(O_2_CMe)_4_(NO_3_)_2_(mepaoH)_2_] (***2***), [Gd_2_(O_2_CMe)_4_(NO_3_)_2_(mepaoH)_2_] (***3***), [Tb_2_(O_2_CMe)_4_(NO_3_)_2_(mepaoH)_2_] (***4***) and [Dy_2_(O_2_CMe)_4_(NO_3_)_2_(mepaoH)_2_] (***5***)

These compounds were prepared in an identical manner with **1**, by using both of the above-mentioned methods, by simply replacing Nd(NO_3_)_3_·6H_2_O with the corresponding hydrated nitrate salts of Eu(III), Gd(III), Tb(III) and Dy(III). Typical yields were ~35% using method (a) and ~25% using method (b). The cited experimental microanalyses values are from samples prepared by method (a). Anal. Calcd. (%) for C_22_H_28_Eu_2_N_6_O_16_ (**2**): C, 28.21; H, 3.02; N, 8.98. Found (%): C, 28.43; H, 2.95; N, 8.67. Anal. Calcd. (%) for C_22_H_28_Gd_2_N_6_O_16_ (**3**): C, 27.90; H, 2.99; N, 8.89. Found (%): C, 27.77; H, 3.04; N, 8.71. Anal. Calcd. (%) for C_22_H_28_Tb_2_N_6_O_16_ (**4**): C, 27.80; H, 2.98; N, 8.84. Found (%): C, 28.12; H, 2.84; N, 9.04. Anal. Calcd. (%) for C_22_H_28_Dy_2_N_6_O_16_ (**5**): C, 27.59; H, 2.95; N, 8.78. Found (%): C, 27.81; H, 3.04; N, 8.70. The IR spectra of **2**–**5** are almost superimposable with the spectrum of **1** with a maximum wavenumber of ±4 cm^−1^.

### 3.4. Synthesis of [Dy_2_(O_2_CMe)_6_(mepaoH)_2_] (***6***)

To a stirred colorless solution of mepaoH (0.136 g, 1.00 mmol) in a solvent mixture comprising MeOH (5 mL) and MeCN (10 mL), solid Dy(O_2_CMe)_3_·4H_2_O (0.215 g, 0.50 mmol) was added. The reaction mixture was stirred under reflux overnight and the obtained slurry was filtered to remove a small quantity of solid. The filtrate was kept in a closed vial at room temperature. X-ray quality, colorless crystals of the product were precipitated within a period of 24 h. The crystals were collected by filtration, washed with cold MeOH (1 mL), MeCN (1 mL) and Et_2_O (3 × 1 mL), and dried in air. The yield was 55% (based on the available Dy^III^). Anal. Calcd. (%) for C_26_H_34_Dy_2_N_4_O_14_: C, 32.81; H, 3.61; N, 5.89. Found (%): C, 32.99; H, 3.44; N, 5.72. Selected IR data (KBr, cm^−1^): 3255 mb, 1593 s, 1540 s, 1465 m, 1418 s, 1128 m, 1085 m, 1023 s, 793 s, 680 s, 410 m.

### 3.5. Conversion of ***6*** to ***5***

A slurry of [Dy_2_(O_2_CMe)_6_(mepaoH)_2_] (**6**) (0.285 g, 0.30 mmol) in MeCN was treated in the hood with 2N HNO_3_ (0.35 mL, 0.70 mmol HNO_3_). The resulting slurry was stirred overnight and the resulting powder was filtered, washed with MeCN (2 × 1 mL) and dried in a vacuum desiccator over P_4_O_10_. The yield was ~70% (based on **6**). Anal. Calcd. (%) for C_22_H_28_Dy_2_N_6_O_16_ (**5**): C, 27.59; H, 2.95; N, 8.78. Found (%): C, 28.04; H, 2.86; N, 8.51. The IR spectrum of the dried white powder is identical with that of the authentic complex **5**.

### 3.6. Single-Crystal X-ray Crystallography

Colorless crystals of **1** (0.08 × 0.09 × 0.30 mm), **5** (0.10 × 0.16 × 0.18 mm) and **6** (0.10 × 0.13 × 0.15 mm) were taken from the mother liquor and immediately cooled to −113 °C (160 K). Diffraction data were collected on a Rigaku R-AXIS SPIDER Image Plate diffractometer (Rigaku Americas Corporation, The Woodlands, TX, USA; European Department at Karlsruhe, Germany) using graphite-monochromated Cu Kα radiation. Data collection (ω-scans) and processing (cell refinement, data reduction, and empirical/numerical absorption correction) were performed using the CrystalClear program package [89]. The structures were solved by direct methods using SHELXS, ver. 2013/1 [90] and refined by full-matrix least-squares techniques of *F*^2^ with SHELXL, ver. 2014/6 [91]. All non-H atoms were refined anisotropically. All H atoms were introduced at calculated positions as riding on their corresponding bonded atoms. Structural plots were drawn using the Diamond 3 program package [92]. Crystallographic data are summarized in Table S1.

Crystallographic data have been deposited with the Cambridge Crystallographic Data Center, Nos 2059448 (**1**), 2059447 (**5**) and 2059449 (**6**). Copies of the data can be obtained free of charge upon application to CCDC, 12 Union Road, Cambridge, CB2 1EZ, UK: Tel.: +(44)-1223-762910; Fax: +(44)-1223-336033; E-mail: deposit@ccdc.cam.ac.uk, or via http://www.ccdc.cam.ac.uk/conts/retrieving.html.

## 4. Concluding Comments and Perspectives

In this work, we have shown that the first use of methyl 2-pyridyl ketoxime (mepaoH) in *homo*metallic Ln(III) chemistry has provided access to complexes containing the {Ln^III^_2_(*η*^1^:*η*^1^:*μ*_2_)_2_(*η*^1^:*η*^2^:*μ*_2_)_2_}^2+^ unit, and one chelating mepaoH ligand and one chelating nitrato (**1–5**) or acetato (**6**) group per metal ion. Although the crystal structures of only three complexes (**1**, **5**, **6**) have been solved by single-crystal X-ray crystallography revealing interesting molecular and supramolecular features, and, despite the fact that we do not have on hand powder XRD patterns for **2**, **3,** and **4**, the spectroscopic and analytical evidence suggests similar structures for the Eu(III), Gd(III), and Tb(III) complexes.

The most salient chemical features of the present work are: (a) the acetato ligands in **1**–**5** arise from the rare Ln^III^-mediated hydrolysis of the solvent used in the reactions (MeCN); and (b) the mixed acetato/nitrato complexes **1**–**5** are the thermodynamically preferred products from the Ln^III^/NO_3_^−^/MeCO_2_^−^/mepaoH reaction systems and that the all-acetato complex [Dy_2_(O_2_CMe)_6_(mepaoH)_2_] (**6**) can be obtained only in the absence of nitrates. We have also demonstrated that the coordination of mepaoH to Eu^III^ leads to a complex that emits red light coming from the enhanced emission of Eu(III). This can be attributed mainly to the shielding of the metal ion from H_2_O ligands (the presence of aqua ligands quenches the emission significantly). The Tb^III^ center in **4** emits in the same region with the ligand and thus the characteristic ^5^*D*_4_→^7^*F*_j_ (j = 3–6) green emission pattern of this metal ion was not observed. In addition, the magnetic study of **3** suggests weak antiferromagnetic intramolecular Gd^III…^Gd^III^ exchange interaction.

We do believe that the research theme presented here is not exhausted of new interesting results. The terminal nitrato (**1**–**5**) or acetato (**6**) groups might have utility as a means to obtain higher-nuclearity neutral or ionic Ln^III^/MeCO_2_^−^/mepaoH clusters and/or polymeric species, by replacing them with bis(bidentate) bridging ligands, e.g., aromatic heterocycles or dicarboxylates; such reactivity could alter the photophysical properties of the resulting complexes. The ability of mepaoH to act as an anionic ligand (mepao^−^) bridging two or three metal ions gives us the stimulus to try to prepare polynuclear Ln^III^/mepao^−^/clusters. In addition, we have been working with the analogues of mepaoH (R = H, Ph, Cl in Figure 1) to study the influence of R on the identity and properties of the products. Last, but not least, inspired by the recent advances in the field of opto-magnetic materials and especially in that of optical thermometers (Single-Molecule Magnets which display thermally controlled luminescence) [93,94], we are investigating the optical/magnetic properties of **4**–**6** and analogous dinuclear Ln(III) complexes with a variety of other neutral 2-pyridyl oximes as capping ligands. Some of the above-mentioned efforts are already well-advanced proving that we have only scratched the surface of the lanthanide(III)/2-pyridyl oxime chemistry, and our results will be reported soon.

## Figures and Tables

**Table 1:** Selected interatomic distances (Å) and angles (°) for complexes **1** and **5** ^a^.

**Interatomic Distances (Å)**		
	1 (Ln = Nd)	5 (Ln = Dy)
Ln1⋯Ln1′	3.877 (1)	3.795 (1)
Ln1-O2	2.555 (6)	2.453 (9)
Ln1-O3	2.577 (5)	2.499 (10)
Ln1-O5	2.426 (5)	2.343 (7)
Ln1-O6′	2.404 (4)	2.312 (8)
Ln1-O7	2.503 (5)	2.421 (8)
Ln1-O8	2.578 (4)	2.522 (8)
Ln1-O8′	2.385 (5)	2.281 (7)
Ln1-N1	2.582 (6)	2.510 (11)
Ln1-N2	2.612 (5)	2.535 (9)
N3-O2	1.273 (8)	1.289 (15)
N3-O3	1.243 (8)	1.266 (13)
N3-O4	1.228 (8)	1.230 (13)
C8-O5	1.265 (8)	1.274 (13)
C8-O6	1.251 (8)	1.253 (14)
C10-O7	1.227 (8)	1.236 (13)
C10-O8	1.289 (9)	1.293 (12)
N1-O1	1.390 (7)	1.386 (13)
**Interatomic Angles (^o^)**		
N1-Ln1-N2	61.1 (2)	62.9 (3)
O2-Ln1-O3	49.8 (2)	52.4 (3)
O7-Ln1-O8	50.7 (2)	52.6 (2)
N2-Ln1-O8′	148.7 (2)	147.1 (3)
O6′-Ln1-O7	91.8 (2)	90.2 (3)
O2-Ln1-O5	120.4 (2)	123.4 (3)
O2-Ln1-O8	154.9 (2)	150.1 (3)
Ln1-O8-Ln1′	102.7 (2)	104.3 (3)

^a^ Symmetry code: (′) = −*x*+2, −*y*+1, −*z*+2.

**Table 2:** Selected interatomic distances (Å) and angles (^o^) for complex **6** ^a^.

Interatomic Distances (Å)		Interatomic Angles (^o^)	
Dy1⋯Dy1′	3.859 (1)	N1-Dy1-N2	61.9 (2)
Dy1-O2	2.413 (5)	O2-Dy1-O3	53.7 (2)
Dy1-O3	2.421 (5)	O7-Dy1-O8	52.0 (2)
Dy1-O5	2.361 (5)	O2-Dy1-O8′	83.8 (2)
Dy1-O6′	2.382 (5)	O3-Dy1-O6′	130.6 (2)
Dy1-O7	2.414 (5)	O5-Dy1-O7	81.4 (2)
Dy1-O8	2.561 (5)	O3-Dy1-O8	143.0 (2)
Dy1-O8′	2.296 (5)	O3-Dy1-O8′	81.4 (2)
Dy1-N1	2.593 (8)	O2-Dy1-O8	147.0 (2)
Dy1-N2	2.525 (7)	O2-Dy1-O5	128.4 (2)
C11-O2	1.259 (9)	N1-Dy1-O5	142.5 (2)
C11-O3	1.254 (9)	N1-Dy1-O8	109.8 (2)
C13-O5	1.242 (9)	N2-Dy1-O3	72.9 (2)
C13-O6	1.279 (9)	N2-Dy1-O5	84.6 (2)
C15-O7	1.252 (8)	N2-Dy1-O8	119.9 (2)
C15-O8	1.262 (8)	Dy1-O8-Dy1′	105.1 (2)
N1-O1	1.395 (9)		

^a^ Symmetry code: (‘) = −*x*, −*y*, −*z*.

## Data Availability

Crystallographic information files are available through the CCDC following the assigned numbers. The data presented here are available on request to the corresponding authors.

## Supplementary Materials

The following are available online, Figure S1: Coordination polyhedra of lanthanide(III) centers in complexes **1**, **5,** and **6**. Figure S2: ORTEP-type plots of the dinuclear molecules in **1** and **5** illustrating the intramolecular H bonds. Figure S3: ORTEP-type plot of the dinuclear molecule in **6** illustrating the intramolecular H bonds. Table S1: Crystallographic data for complexes **1**, **5,** and **6**. Table S2: CShM values for the potential polyhedra of the lanthanide(III) centers in **1**, **5,** and **6**. Table S3: Distances and angles of the intra-and intermolecular H-bonding interactions in the crystal structures of complexes **1** and **5**. Table S4: Distances and angles of the intra- and intermolecular H-bonding interactions in the crystal structure of complex **6**.

## Sample Availability

Samples of the compounds presented in this study are available on request to the corresponding authors.
