# Supplementary material for: Dinuclear Lanthanide(III) Complexes from the Use of Methyl 2-Pyridyl Ketoxime: Synthetic, Structural, and Physical Studies
Source: Molecules. 2021 Mar 15;26(6):1622. doi: 10.3390/molecules26061622 (PMC7999197; doi:10.3390/molecules26061622)
Supplement: Supplementary file 1 [file molecules-26-01622-s001.pdf]

molecules MDPI

Supplementary Materials

Dinuclear Lanthanide(III) Complexes from the Use of Methyl 2-pyridyl Ketoxime: Synthetic, Structural and Physical Studies

Christina D. Polyzou [1], Helen Nikolaou [1], Catherine P. Raptopoulou [2], Konstantis F. Konidaris [3], Vlasoula Bekiari [4,*], Vassilis Psycharis [2,*] and Spyros P. Perlepes [1,5,*]

1 Department of Chemistry, University of Patras, 26504 Patras, Greece; christipoche@gmail.com (C.D.P.); eleni.nikolaou-2@postgrad.manchester.ac.uk (H.N.)
2 Institute of Nanoscience and Nanotechnology, NCSR "Demokritos", 15310 Aghia Paraskevi Attikis, Greece; c.raptopoulou@inn.demokritos.gr (C.P.R.)
3 Department of Pharmacy, University of Patras, 26504 Patras, Greece; konidaris@upatras (K.F.K.)
4 Department of Crop Science, University of Patras, 30200 Messolonghi, Greece; bbekiari@upatras.gr (V.B.)
5 Foundation for Research and Technology – Hellas (FORTH), Institute of Chemical Engineering Sciences (ICE – HT), Platani, B.O. Box 1414, 26504 Patras, Greece
* Correspondence: bbekiari@upatras.gr (V.B.); v.psycharis@inn.demokritos.gr (V.P.); perlepes@patreas.upatras.gr (S.P.P.); Tel: +30-26130-58240 (V.B.); +30-210-6503346 (V.P.); +30-2610-996730 (S.P.P.)

Table S1. Crystallographic data and structural refinement parameters for complexes **1**, **5** and **6**.

**Parameter**	**1**	**5**	**6**
Formula	$C_{22}H_{28}N_6Nd_2O_{16}$	$C_{22}H_{28}N_6Dy_2O_{16}$	$C_{26}H_{34}Dy_2N_4O_{14}$
Formula weight	920.98	957.50	951.57
Crystal color	colorless	colorless	colorless
Crystal size, mm	0.30 x 0.09 x 0.08	0.18 x 0.16 x 0.10	0.15 x 0.13 x 0.10
Crystal system	triclinic	triclinic	monoclinic
Space group	$P\bar{1}$	$P\bar{1}$	$P2_1/c$
Temperature, K	160	160	160
Radiation, Å	Cu Kα, 1.54178	Cu Kα, 1.54178	Cu Kα, 1.54178
a, Å	8.1064(2)	8.0902(5)	8.7141(1)
b, Å	8.3855(2)	8.3503(3)	12.9093(2)
c, Å	12.0672(2)	11.8921(6)	15.3750(2)
α, °	98.087(1)	98.748(1)	90.0(1)
β, °	99.681(1)	100.335(1)	107.586(1)
γ, °	99.893(1)	99.857(1)	90.0(1)
Volume, $Å^3$	784.34(3)	764.70(7)	1648.75(4)
Z	1	1	2
Calculated density, $g{\cdot}cm^{-3}$	1.950	2.079	1.917
Absorption coefficient, mm^{-1}	25.69	26.59	24.59
θ_{min}-θ_{max}, °	7.0-65.0	7.0-65.0	6.7-65.0
Reflections collected/unique (R_{int})	12135/2445 (0.078)	8564/2350 (0.113)	21531/2879 (0.076)
Reflections with I>2σ(I)	2374	1796	2601
Refined parameters/restrains	212/0	212/0	212/0
R_1[a][I>2σ(I)], R_1[a] (all data)	0.0462, 0.0469	0.0875, 0.0996	0.0523, 0.0552
wR_2[b][I>2σ(I)], wR_2 (all data)	0.1158, 0.1170	0.1728, 0.1895	0.1324, 0.1351
Goodness-of-fit on F^2	1.03	1.15	1.06
Largest differences peak and hole (e $Å^{-3}$)	2.12/-2.07	2.46/-1.72	1.64/-2.41

[a] $R_1 = \Sigma(|F_o|-|F_c|) / \Sigma(|F_o|)$. [b] $wR_2 = \{\Sigma[w(F_o^2-F_c^2)^2] / \Sigma[w(F_o^2)^2]\}^{1/2}$, $w = 1[\sigma^2(F_o^2) + (aP)^2 + bP]$, where $P = [\max(F_o^2, 0) + 2F_c^2]/3$ (a = 0.0782 and b = 0.9824 for **1**; a = 0.0713 and b = 0.03264 for **5**; a = 0.724 and b = 10.8106 for **6**).

Table S2. Continuous Shape Measures (CShM) values[a] for the potential coordination polyhedra of the crystallographically independent Ln^{III} centers in the structures of complexes **1**, **5** and **6**.

**Ideal Coordination Polyhedron**	**1**	**5**	**6**
Enneagon (EP-9)	34.398	34.902	34.011
Octagonal pyramid (OPY-9)	21.909	22.085	22.489
Heptagonal bipyramid (HBPY-9)	17.795	17.915	17.333
Johnson triangular cupola (JTC-9)	15.576	15.552	14.592
Capped cube (JCCU-9)	11.176	10.707	9.358
Spherical-relaxed capped cube (CCU-9)	9.344	9.544	8.647
Capped square antiprism (JCSAPR-9)	2.865	2.219	2.620
Spherical-capped square antiprism (CSAPR-9)	2.110	1.598	1.992
Tricapped trigonal prism (JTCTPR-9)	3.584	3.190	3.117
Spherical tricapped trigonal prism (TCTPR-9)	2.754	2.405	2.517
Tridiminished icosahedron (JTDIC-9)	13.286	13.267	12.424
Hula-hoop (HH-9)	9.232	9.620	9.902
Muffin (MFF-9)	**1.664**	**1.316**	**1.578**

[a]The polyhedron with the smallest CShM value (in bold) is the real coordination polyhedron of the Ln/Ln1′ center for the complex.

Table S3. Intra- and intermolecular H-bonding interactions (distances in Å and angles in °) in the crystal structures of the isomorphous complexes **1** and **5**. [a,b]

**D-H···A**	**D-H**	**H···A**	**D···A**	**D-H···A**
		Complex **1**		
O1-H(O1)···O5	0.84	2.01	2.734(7)	145
C7-H(C7)···O6′	0.95	2.57	3.261(8)	129
C4-H(C4)···$O7^{i}$	0.95	2.49	3.376(9)	155
C9-H_B(C9)···$O4^{ii}$	0.98	2.43	3.383(11)	164
C11-H_B(C11)···$O2^{ii}$	0.98	2.60	3.469(11)	149
C1-H_A(C1)···O1′′	0.98	2.52	3.392(9)	148
C1-H_B(C1)···O4′′′	0.98	2.47	3.319(9)	145
		Complex **5**		
O1-H(O1)···O5	0.84	2.02	2.690(12)	136
C7-H(C7)···O6′	0.95	2.44	3.107(14)	127
C4-H(C4)···$O7^{i}$	0.95	2.51	3.391(17)	155
C9-H_B(C9)···$O4^{ii}$	0.98	2.42	3.348(15)	157

C11-H$_B$(C11)···O2^{ii}	0.98	2.56	3.434(18)	148
C1-H$_A$(C1)···O1''	0.98	2.55	3.402(15)	146
C1-H$_B$(C1)···O4'''	0.98	2.59	3.35(2)	134

[a] D = donor, A = acceptor. [b] Symmetry codes: (') = -*x*+2, -*y*+1, -*z*+2; ('') = -*x*+1, -*y*+1, -*z*+1; (''') = -*x*+1, -*y*, -*z*+1; (^{i}) = -*x*+2, -*y*+1, -*z*+1; (^{ii}) = *x*, *y*+1, *z*.

Table S4. Intra- and intermolecular H-bonding interactions (distances in Å and angles in °) in the crystal structure of **6**. [a,b]

**D-H···A**	**D-H**	**H···A**	**D···A**	**D-H···A**
O1-H(O1)···O6'	0.84	2.03	2.603(8)	125
C7-H(C7)···O5	0.95	2.52	3.185(12)	127
C16-Hc(C16)···O2^{ii}	0.98	2.46	3.412(9)	164
C4-H(C4)···O3''	0.95	2.35	3.114(11)	137
C6-H(C6)···O2'''	0.95	2.44	3.200(10)	137
C14-H$_C$(C14)···O7^{i}	0.98	2.58	3.521(9)	162

[a] D = donor, A = acceptor. [b] Symmetry codes: (') = -*x*, -*y*, -*z*; ('') = -*x*+1, *y*+1/2, -*z*+1/2; (''') = *x*+1, *y*, *z*; (^{i}) = -*x*+*1*, -*y*, -*z*; (^{ii}) = *x*, -*y*+1/2, *z*-1/2.

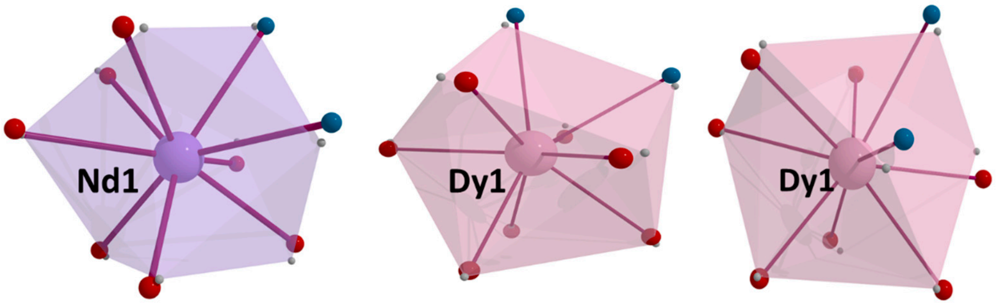

Figure S1. The muffin-type coordination polyhedra of Ln1/Ln1′ in complexes **1** (left), **5** (middle) and **6** (right). The plotted polyhedra with the small spheres as vertices are the best-fit polyhedra created by using the program SHAPE.

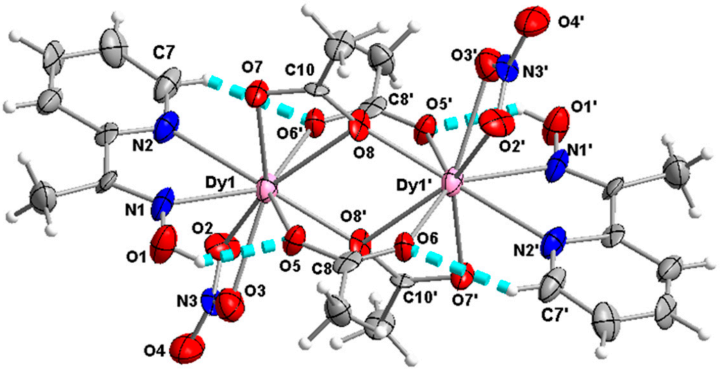

(a)

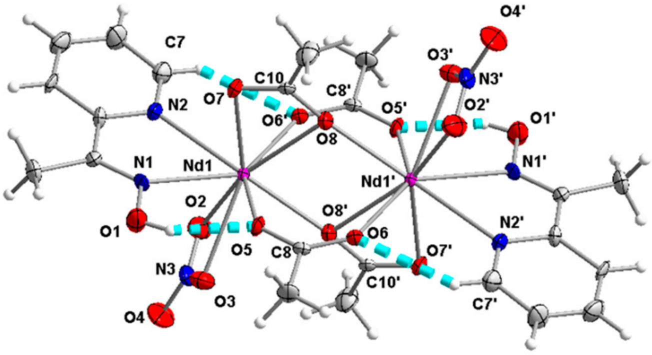

(b)

Figure S2. Intramolecular H bonds (dashed cyan lines) in the dinuclear complexes $[Dy_2(O_2CMe)_4(NO_3)_2(mepaoH)_2]$ (**5**, **a**) and $[Nd_2(O_2CMe)_4(NO_3)_2(mepaoH)_2]$ (**1**, **b**). The ellipsoids are drawn at the 50% thermal probability level. Symmetry code: (′) = -*x*+2, -*y*+1, -*z*+2.

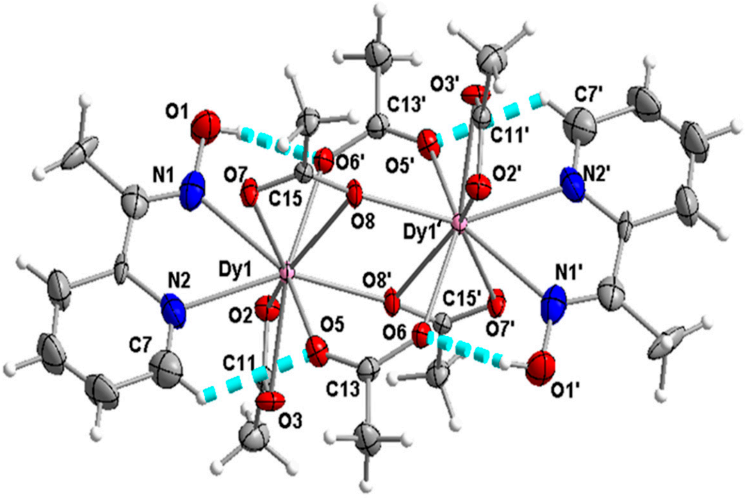

Figure S3. Intramolecular H bonds (dashed cyan lines) in the dinuclear complex $[Dy_2(O_2CMe)_6(mepaoH)_2]$ (**6**). The ellipsoids are drawn at the 50% thermal probability level. Symmetry code: (‘) = -*x*, -*y*, -*z*.
